# Establishment of a typing model for diffuse large B-cell lymphoma based on B-cell receptor repertoire sequencing

**DOI:** 10.1186/s12885-021-08015-z

**Published:** 2021-03-22

**Authors:** Wenhua Jiang, Hailong Wang, Shiyong Zhou, Guoqing Zhu, Mingyou Gao, Kuo Zhao, Limeng Zhang, Xiaojing Xie, Ning Zhao, Caijuan Tian, Zhenzhen Zhang, Fang Yan, Yi Pan, Pengfei Liu

**Affiliations:** 1 Department of Radiotherapy, The Second Hospital of Tianjin Medical University, Tianjin, 300211 China; 2 Department of Oncology, Tianjin Academy of Traditional Chinese Medicine Affiliated Hospital, No.354 Beima Road, Hongqiao District, Tianjin, 300120 China; 3 Department of Lymphoma, Sino-US Center of Lymphoma and Leukemia, National Clinical Research Center for Cancer, Key Laboratory of Cancer Prevention and Therapy, Tianjin’s Clinical Research Center for Cancer, Tianjin Medical University Cancer Institute and Hospital, Tianjin, 300060 China; 4 State Key Laboratory of Experimental Hematology, National Clinical Research Center for Blood Diseases, Institute of Hematology & Blood Diseases Hospital, Chinese Academy of Medical Sciences & Peking Union Medical College, Tianjin, 300020 China; 5 Department of Oncology, National Clinical Research Center for Cancer, Key Laboratory of Cancer Prevention and Therapy of Tianjin, Tianjin Medical University Cancer Institute and Hospital, Tianjin, 300060 China; 6 Tianjin Marvel Medical Laboratory, Tianjin Marvelbio Technology Co., Ltd, Tianjin, 300381 China; 7 Department of Pathology, National Clinical Research Center for Cancer, Key Laboratory of Cancer Prevention and Therapy of Tianjin, Tianjin Medical University Cancer Institute and Hospital, Huanhu West Road, Tiyuanbei, Hexi District, Tianjin, 300060 China

**Keywords:** Diffuse large B-cell lymphoma, B-cell receptor repertoire, Typing, Prognosis

## Abstract

**Background:**

The purpose of this study was to construct a new typing model for diffuse large B-cell lymphoma (DLBCL) patients based on the B-cell receptor (BCR) and explore its potential molecular mechanism.

**Methods:**

BCR repertoire sequencing and whole-exome sequencing were performed on formalin-fixed paraffin-embedded samples from 12 DLBCL patients. Subsequently, a typing model was built with cluster analysis, and prognostic indicators between the two groups were compared to verify the typing model. Then, mutation and bioinformatics analyses were conducted to investigate the potential biomarkers of prognostic differences between the two groups.

**Results:**

Based on BCR sequencing data, we divided patients into two clusters (cluster 1 and cluster 2); this classification differed from the traditional typing method (GCB and non-GCB), in which cluster 1 included some non-GCB patients. The progression-free survival (PFS), overall survival (OS), metastasis and Shannon diversity index of IGH V-J and survival after chemotherapy were significantly different (*P* < 0.05) between the two clusters, but no statistical significance was found between the GCB and non-GCB groups. The mutation status of 248 genes was significantly different between cluster 1 and cluster 2. Among them, *FTSJ3, MAGED2*, and *ODF3L2* were the specific mutated genes in all patients in cluster 2, and these genes could be considered critical to the different prognoses of the two clusters of DLBCL patients.

**Conclusion:**

We constructed a new typing model of DLBCL based on BCR repertoire sequencing that can better predict the survival time after chemotherapy. *FTSJ3, MAGED2*, and *ODF3L2* may represent key genes for the difference in prognosis between the two clusters.

## Background

Diffuse large B-cell lymphoma (DLBCL) is a moderately aggressive lymphoma originating from B cells and is the most common type of non-Hodgkin lymphoma (NHL). DLBCL is a heterogeneous disease due to its clinical manifestations and morphological and genetic characteristics [1]. DLBCL accounts for approximately 30% of adult NHL in developed countries, while the incidence rate in developing China is estimated to be higher [2, 3]. Typically, patients with DLBCL are divided into two subtypes according to gene expression profiling (GEP) technology, namely germinal centre B cell (GCB) type and activated B cell (ABC) type. Given that mutations in Toll-like receptor (TLR) and B-cell receptor (BCR) signalling molecules play a key role in the pathogenesis and progression of DLBCL, various downstream targets are being developed to obtain clinical therapeutic benefits [4].

The immunohistochemistry classification method is the gold standard for DLBCL typing. CD10, Bcl-6, MUM1, GCET1, and FOXP1 are used as markers to divide DLBCL into 2 subtypes: GCB and non-GCB. However, a number of studies have found that DLBCL histological morphology, immunophenotype, genetic characteristics, and clinical incidence vary greatly among different cases. The heterogeneity of DLBCL and the limitations of the international prognostic index (IPI) in assessing prognosis indicate that DLBCL may have different pathological subtypes. Therefore, accurate classification of DLBCL and clarification of its characteristics are particularly important in the exploration of new therapeutic schemes.

B cells are key participants in the adaptive immune system. These cells mediate fluid immunity through the production of antibodies, thus protecting the body from infection. Each B cell expresses a unique membrane-binding antibody or B-cell receptor (BCR). Other parts of the immune system, such as antigen-presenting and follicular helper T (Tfh) cells, also need to function effectively and interact with B cells to produce a variety of functional BCR components stimulated by antigens [5]. The various BCRs produced in individuals must be sufficient for the immune system to recognize a large number of different antigens. This large variation is produced by DNA recombination of different BCR variable (V), diversity (D) and joining (J) genes (called VDJ recombination) [5]. These genes are highly polymorphic and constitute the basis of BCR allele diversity in individuals. During B–cell mutation, V, D and J (or V and J in the light chain) gene fragments are recombined to form functional BCR proteins. The diversity of V/(D)/J alleles and V-(D)-J combinations yield a highly diversified BCR repertoire [6].

BCR diversity comprises one of the core components of the complicated immune system and serves as a pivotal defensive component to protect the body against invading viruses, bacteria, etc. [7]. The diversity of the BCR repertoire may vary greatly among different health conditions. Although this type of antigenic specificity ensures protection against infectious diseases, the imbalance between exogenous and autoantigen discrimination may lead to immunodeficiency or even malignant tumours [8]. Under different phenotypic statuses, BCRs may change dynamically. Even under the same phenotype, the BCR repertoire of different patients exhibit great differences. BCR sequencing provides an opportunity to monitor the evolution of the B-cell response by describing the diversity of BCR gene sequences. Researchers have demonstrated the utility of BCR sequencing in adaptive immune responses [9–11]. With the development of next-generation sequencing (NGS) technology, BCR sequencing has the potential to reliably quantify all aspects of the adaptive immune response. BCR repertoire sequencing in DLBCL patients may help us to understand the developmental mechanism of different DLBCLs and predict the prognosis of patients. However, most studies based on BCR sequencing have focused on the use of the human immunoglobulin heavy chain (IGH) V-(D)-J gene as a measure of BCR diversity and clonal evolution to describe B-cell responses in health and disease [12].

Here, we constructed a typing model in DLBCL patients based on BCR repertoire sequencing to contribute to accurate prognosis prediction to achieve precision medicine in DLBCL.

## Methods

### Patients and samples

A total of 12 patients with DLBCL in our hospital who had not received immunotherapy were enrolled in this study. Formalin-fixed paraffin-embedded (FFPE) tumour samples from all patients were collected. The clinical characteristics of these patients are listed in Table 1.

**Table 1:** Clinical characteristics of patients

Parameters	Group	n (%)	Parameters	Group	n (%)
Sex	male	8 (66.67)	KPS	≤ 80	3 (25.00)
	female	4 (33.33)		80–90	8 (66.67)
Age	≤ 60	6 (50.00)		> 90	1 (8.33)
	> 60	6 (50.00)	Recurrence	no	12 (100)
Stage	I	1 (8.33)		yes	0 (0)
	II	5 (41.67)	Metastasis	no	8 (66.67)
	III	0 (0)		yes	4 (33.33)
	IV	6 (50.00)	OS	≤ 24 months	8 (66.67)
Infection	HAV	12 (100)		> 24 months	4 (33.33)
	HBV	9 (75.00)	PFS	≤ 6 months	2 (16.67)
	HCV	0 (0)		> 6 months	2 (16.67)
Surgery	no	0 (0)		none	8 (66.67)
	yes	12 (100)			

### DNA isolation, CDR3 amplification and sequencing library construction

Genomic DNA was extracted from FFPE samples using an AllPrep FFPE DNA Extraction Kit (Qiagen, Hildon, Germany) according to the manufacturer’s instructions. BCR complementarity determining region 3 (CDR3) was amplified using a Multiplex PCR kit (Qiagen, Hilden, Germany), and a 100 to 200-bp fragment was selected and purified using a QIAquick gel purification kit (Qiagen, Hilden, Germany). The DNA was then subjected to end repair. The A tail was added, ligated, adapte, subsequently amplified by PCR and further purified with Agencourt AMPure XP beads (Agencourt Biosciences, Beverly, MA, USA). The sequencing library was quantified with Invitrogen Quibit 2.0 (Invitrogen, California, USA).

### Immunohistochemistry

Immunohistochemistry was performed using the EnVision two-step method. TBS was used as a blank control instead of a primary antibody, and the corresponding positive sections were used as a positive control. The pathologists read the film in a double-blind fashion, and the average value was used to calculate the results. The results were qualitatively judged, and the result was considered positive when > 20% of tumour cells appeared clear granular brown in colour. CD10-positive staining was located in the cell membrane, whereas BCL-6- and MUM1-positive staining was located in the nucleus. The criteria for determining the subtypes by immunohistochemistry were as follows: CD10(+) and CD10(−)BCL-6(+)MUM-1(−) were classified as GCB type; CD10(−)BCL-6(−) and CD10(−)BCL-6(+)MUM-1(+) were classified as non-GCB type.

### BCR repertoire sequencing

BCR repertoire sequencing was performed using the Illumina HiSeq 2500 platform with a read length of 2 × 150 bp, and each sample produced at least 3G of data. After filtering the low-quality sequences, the original data were converted to raw paired-end sequence reads. The sequences of the IGH V, IGH J and CDR3 regions were identified using BLAST version 2.2.21 in the international ImMunoGeneTics (IMGT) information system (http://www.imgt.org/). The cluster analysis for IGH V and IGH J region sequences in BCR of 12 patients were conducted with the pheatmap package (version 1.0.12) using hierarchical clustering method. The Shannon index was calculated using the following formula:

$$ \mathrm{H}=\hbox{-} \sum \limits_{\mathrm{i}=1}^{\mathrm{s}}\left({p}_i{\log}_2{p}_i\right) $$

where s is the count of V/J sequences, and pi is the ratio of the i-th operational taxonomic unit.

### Whole-exome sequencing

DNA from FFPE samples was extracted using the AllPrep-FFPE-DNA Extraction Kit (Qiagen). Library preparation for each sample was performed according to the instructions provided by the manufacturer. Briefly, 3 μg of high-quality genomic DNA was randomly cut into 150 to 200-bp fragments. Agencourt AMPure XP beads (Agencourt Biosciences, Beverly, Massachusetts, USA) were used for size selection, and PCR amplification was then performed. The whole-exome sequences were enriched using the SureSelect Human All Exon Kit V5 (Agilent) and pooled and sequenced using the Illumina HiSeq Xten platform.

### Mutation and functional enrichment analyses

The sequencing data were mapped to the hg19 reference genome using Burrows-Wheeler-Aligner software (http://bio-bwa.sourceforge.net) for tumour-specific somatic mutation detection. Gene Ontology (GO) terms and Kyoto Encyclopedia of Genes and Genomes (KEGG) pathway enrichment analyses were performed using the KEGG Orthology Based Annotation System (KOBAS; http://kobas.cbi.pku.edu.cn/kobas3), and *P* < 0.05 was the cut-off criterion.

### Statistical analysis

Two-sample Wilcoxon tests were used to compare the differences of all indexes (V/J pairs, Shannon index, survival time, metastasis, recurrence, etc.) between groups (package stats version 3.6.2). Survival analysis was performed using the log-rank test (package survival version 3.1–8) and mapped with the survivor package (version 0.4.6). Spearman sequential correlation analysis was used for correlation analysis, and the R package psych (version 1.9.12.31) was employed. All continuous variable data were tested for a normal distribution.

## Results

### New typing model construction based on cluster analysis

**Fig. 1:**
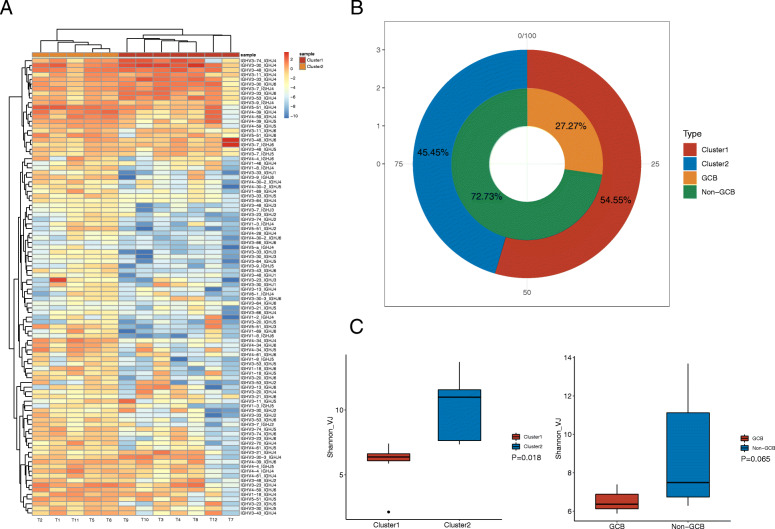
Typing model construction in DLBCL patients through cluster analysis. **a** The patients were clustered into two groups according to V-J pairs usage; **b** The proportion of patients in each group compared with traditional classification; **c** Comparison of Shannon index between different types of groups. DLBCL, diffuse large B-cell lymphoma

IGH CDR3 contains 48 V and 6 J regions, which determine the specificity and partly reflect the diversity of BCR. After standardizing the percentage content of each V-J sequence of each patient, we performed cluster analysis and divided the patients into two groups based on the content of the V-J sequence in each patient (cluster 1 and cluster 2, Fig. 1a). These patients were also classified according to the traditional method of immunohistochemistry results (GCB and non-GCB, Table 2), and the proportions of the two classification methods were compared. All GCB patients were clustered into cluster 1, cluster 2 contained all non-GCB patients, and some non-GCB patients were clustered into cluster 1 (Fig. 1b).

**Table 2:** Immunohistochemistry results of all patients

Subtype	Cluster	CD10	BCL-6	MUM-1
GCB	Cluster 1	+	+	–
GCB	Cluster 1	+	+	+
GCB	Cluster 1	+	+	+
Non-GCB	Cluster 2	–	+	+
Non-GCB	Cluster 2	–	+	+
Non-GCB	Cluster 2	–	+	+
Non-GCB	Cluster 2	–	+	+
Non-GCB	Cluster 2	–	–	Null
Non-GCB	Cluster 1	–	–	–
Non-GCB	Cluster 1	–	+	+
Non-GCB	Cluster 1	–	–	Null
Not identified	Cluster 1	Null	Null	Null

Subsequently, we compared the V, J, and V-J sequences of clusters 1 and 2, and identified sequences with significant differences. Table 3 lists the different sequences of V, J, and the top six V-J pairs between the two groups. The comparison was also conducted using the GCB and non-GCB groups (Table 4). Only IGH V3-33_IGH J2 and IGH V5-a_IGH J4 coexisted in the two groups of different sequences, indicating that the distribution of V-J sequences differed greatly between the two typing methods in DLBCL patients.

**Table 3:** The differentially usage of V, J sequences, and top 6 V-J pairs between cluster 1 and cluster 2

Sequence	Cluster 1 (%)	Cluster 2 (%)	*P* value
IGH J3	0.028 (0.0003, 3.847)	1.299 (0.482, 17.882)	0.030
IGH V3–9	0.617 (0.043, 1.858)	3.037 (2.135, 4.120)	0.003
IGH V3–43	0.038 (0.001, 0.839)	1.835 (0.771, 2.742)	0.005
IGH V4–39	2.319 (0.014, 6.323)	8.643 (3.359, 15.167)	0.010
IGH V1–8	0.009 (0.0008, 1.364)	0.830 (0.267, 1.661)	0.030
IGH V5–51	2.989 (0.040, 11.652)	10.662 (7.124, 18.537)	0.030
IGH V3–23	1.930 (0.008, 6.065)	5.776 (2.940, 20.019)	0.048
IGH V3-33_IGH J2	0.003 (3.6E-05, 0.022)	0.122 (0.053, 1.274)	0.003
IGH V3-33_IGH J3	0.0002 (2.92E-05, 0.003)	0.048 (0.003, 0.175)	0.003
IGH V3–43_IGH J6	0.001 (0.0006, 0.003)	0.127 (0.016, 0.479)	0.003
IGH V3-64_IGH J5	0.001 (4.44E-05, .003)	0.012 (0.004, 0.034)	0.003
IGH V4-34_IGH J4	0.017 (0.002, 0.089)	1.504 (0.591, 4.284)	0.003
IGH V5-a_IGH J4	0.0008 (2.96E-05, 0.004)	0.005 (0.004, 0.240)	0.003

**Table 4:** The differentially V, J sequences, and V-J pairs between GCB and non-GCB groups

Sequence	GCB (%)	Non-GCB (%)	*P* value
IGH V3–7	7.714 (5.309, 7.769)	3.776 (0.844, 6.032)	0.024
IGH V4–61	0.215 (0.067, 0.563)	0.900 (0.474, 3.428)	0.024
IGH V5-a	0.000 (0.0003, 0.002)	0.004 (0.0008, 0.240)	0.048
IGH V3-30_IGH J6	5.094 (4.581, 6.220)	1.383 (0.575, 4.396)	0.012
IGH V3–7_IGH J5	1.104 (0.829, 1.552)	0.351 (0.008, 0.757)	0.012
IGH V4–39_IGH J6	0.067 (0.005, 0.174)	1.564 (0.549, 2.377)	0.012
IGH V4-34_IGH J6	0.003 (0.001, 0.004)	0.663 (0.004, 1.885)	0.024
IGH V4–61_IGH J4	0.017 (0.008, 0.150)	0.340 (0.139, 1.498)	0.024
IGH V3-33_IGH J2	0.003 (3.6E05, 0.005)	0.081 (0.003, 1.274)	0.048
IGH V3-33_IGH J5	0.021 (0.017, 0.023)	0.110 (0.020, 0.977)	0.048
IGH V3-48_IGH J4	5.694 (5.484, 9.900)	4.444 (0.674, 6.797)	0.048
IGH V3–7_IGH J4	4.460 (3.663, 6.237)	2.265 (0.457, 4.709)	0.048
IGH V5-a_IGH J4	0.0007 (0.0003, 0.002)	0.0036 (0.0008, 0234)	0.048

The diversity of BCR sequences reflects the immune function of the body, which varies greatly in different populations. The results of the immune diversity comparison are shown in Fig. 1c. After cluster typing, the Shannon index of cluster 1 was significantly reduced compared with that of cluster 2 (*P* = 0.018), suggesting that patients in cluster 2 had a richer immune system. Although the Shannon index was higher in the non-GCB group, no statistical difference was note between the GCB and non-GCB groups (Fig. 1c).

### The cluster typing model showed better prognostic prediction ability especially after chemotherapy

**Fig. 2:**
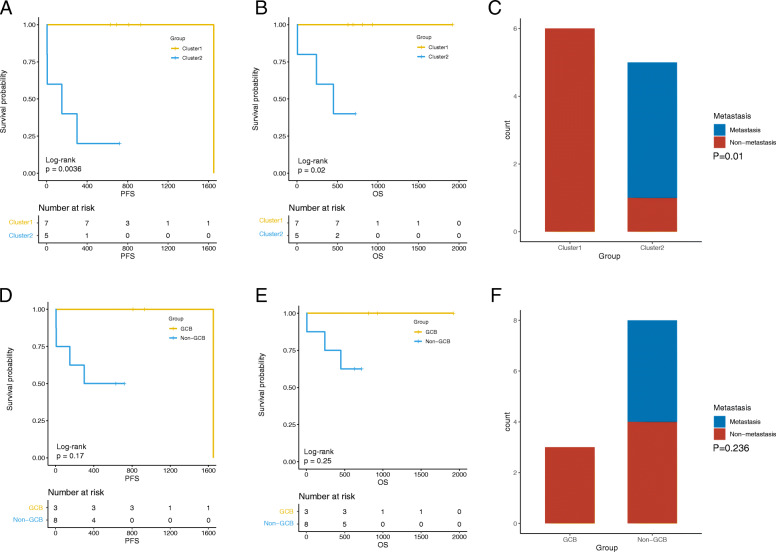
Comparison of prognosis and survival outcomes of DLBCL patients between different groups after cluster and traditional typing. The PFS (a) and OS (**b**) of patients in cluster 1 were significantly higher than patients in cluster 2, and number of patients with metastasis (**c**) was markedly lower in cluster 1; while PFS (**d**), OS (**e**) and metastasis (**f**) were no statistical difference between GCB and non-GCB groups. DLBCL, diffuse large b-cell lymphoma; PFS: progression-free survival; OS, overall survival; GCB, germinal center B cell-like lymphoma

After follow-up for up to 64 months, the prognosis of patients after cluster classification and traditional classification were compared, and the results are shown in Fig. 2. After cluster analysis, the patients who were clustered into cluster 1 had longer PFS (median PFS: 1650 vs. 150 [0, 457.031], *P* = 0.004, Fig. 2a) and OS (median OS: none vs. 450 [0,900.885], *P* = 0.020, Fig. 2b) and lower metastasis rates (0 vs. 80%, *P* = 0.010, Fig. 2c), and the difference was statistically significant. In comparison, although the GCB group exhibited an increased survival time (Fig. 2d, e) and better prognosis (0 vs. 50%, Fig. 2f) than the non-GCB group, no significant difference was found in our study (*P* > 0.05).

**Fig. 3:**
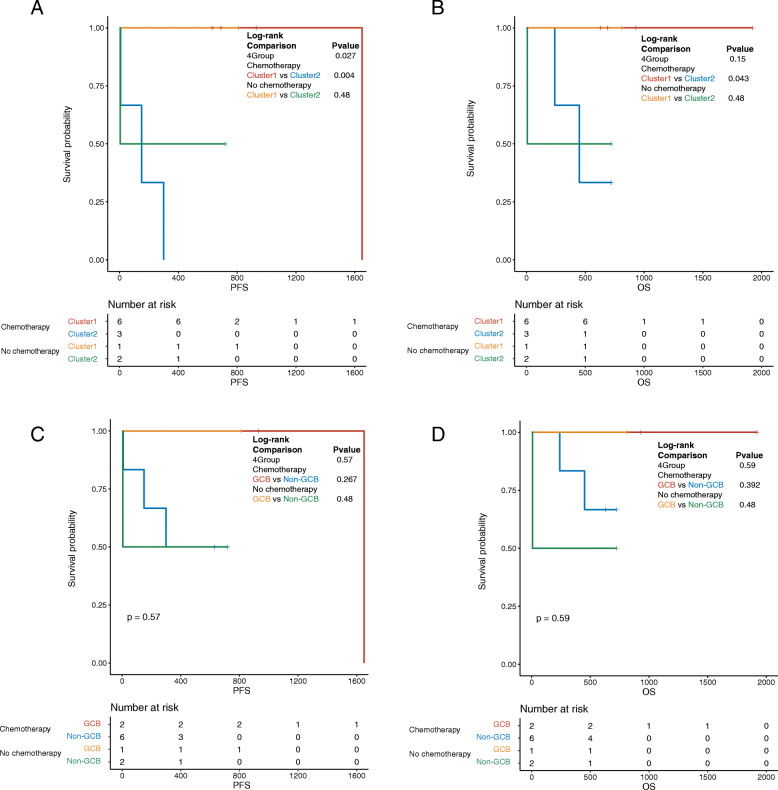
Effect of chemotherapy on survival of DLBCL patients in different groups. **a-b** PFS and OS of patients with or without chemotherapy in cluster 1 and cluster 2; C-D: PFS and OS of patients with or without chemotherapy in GCB and non-GCB groups. DLBCL, diffuse large b-cell lymphoma; PFS: progression-free survival; OS, overall survival; GCB, germinal center B cell-like lymphoma

Next, we divided each group of patients into two subgroups according to whether they had received chemotherapy and observed the effect of chemotherapy on the survival of patients in each group. The results were illustrated in Fig. 3. Among the patients who received chemotherapy, patients who clustered into cluster 1 had significantly higher PFS (*P* = 0.004, Fig. 3a) and OS (*P* = 0.043, Fig. 3b) than those in cluster 2, and no significant difference was noted among the non-chemotherapy patients. In addition, significant differences in PFS were noted among the four subgroups (*P* = 0.027, Fig. 3a). In contrast, after traditional typing, no difference was found in the OS and PFS of patients in the GCB group compared with the non-GCB group (Fig. 3c, d) regardless of chemotherapy. The results revealed that compared with traditional typing methods, our model can predict the prognosis of patients more accurately after chemotherapy and has a stronger guiding role in the clinical treatment of DLBCL.

### Identification of specific mutated genes between the two clusters

**Fig. 4:**
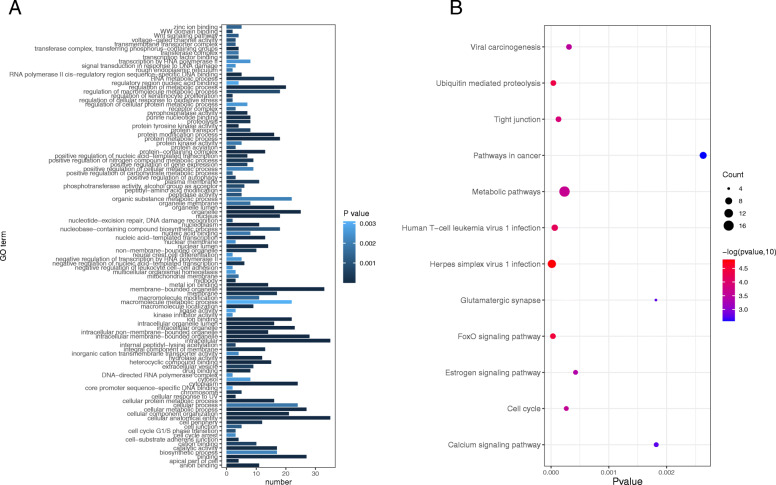
GO terms (**a**) and KEGG pathways (**b**) enriched by differentially mutated genes in cluster 1 and cluster 2. GO, Gene Ontology; KEGG, Kyoto Encyclopedia of Genes and Genomes

A total of 248 genes with significant differences in mutation status were noted between patients in cluster 1 and cluster 2. These genes were enriched in 103 GO terms (Fig. 4a), including 35 cellular components, 42 biological processes, and 26 molecular functions. In addition, these genes were also enriched in 12 KEGG pathways (Fig. 4b). Figure 4 shows that more genes were significantly enriched in GO terms of membrane-bound organelles, intracellular and cellular anatomical entity as well as in KEGG pathways of metabolic pathways and pathways in cancer. Furthermore, *FTSJ3, MAGED2*, and *ODF3L2* were mutated in all cluster 2 patients but not in all cluster 1 patients.

## Discussion

In this study, we developed a novel typing model of DLBCL according to IGH V and J regions from the perspective of BCR repertoire sequencing and compared the predictive ability of DLBCL with traditional typing methods in prognosis and survival. Our new typing model is betterthan traditional classification in predicting the survival time and prognosis of patients (Fig. 2). Our model combined with the traditional method may improve the accuracy of the prognosis of lymphoma. In addition, based on our model, the survival time of the two groups after chemotherapy was significantly different (*P* = 0.043, Fig. 3). None of the patients in cluster 1 had metastasis, whereas 80% of the patients in cluster 2 had metastasis (Fig. 2c). At present, the National Comprehensive Cancer Network (NCCN) considers rituximab combined with cyclophosphamide + doxorubicin + vincristine + prednisone (R-CHOP) as the gold standard for DLBCL treatment, but approximately 1/3 of patients exhibit disease progression after first-line treatment [13]. The results of this study preliminarily indicate that our typing method can predict the prognosis of patients after chemotherapy to assist doctors in determining the treatment plan in clinical practice.

Another interesting finding of our study is that cluster 1 patients had a higher survival time and lower metastasis rate, but their diversity was lower than that of cluster 2 patients (Fig. 1c). BCR has rich diversity, which is mainly reflected in the diversity of heavy chains, light chains and their pairing combinations [14]. Healthy organisms can respond to almost all foreign bodies that invade. Therefore, increased BCR diversity typically indicates better immune status. However, our results are contrary, which may be due to the following reasons. Our study was performed in patients with DLBCL, which is a B-cell lymphoma, and its different subtypes and phenotypes may be closely related to BCR diversity. In addition, therapies, such as chemotherapy and targeted therapy, target B cells in DLBCL patients. Previous studies have found that after treatment, the immune function of NHL patients is reduced. Therefore, we hypothesise that the activity and diversity of B cells may be reduced to varying degrees. In addition, the better the curative effect, the greater the weakening of BCR diversity. The follow-up survival analysis after chemotherapy also confirmed our conjecture that patients with decreased immune diversity in the V-J region of BCRs experienced improved chemotherapy effects and longer survival times after treatment. To the best of our knowledge, at the time of writing, there has been no study on the diversity of BCR repertoires in DLBCL. Further investigation is warranted to verify and advance this finding.

Furthermore, we screened the specific mutated genes in the two clusters to explore the mechanism of the new typing method at the molecular level. Targeted NGS sequencing revealed that *FTSJ3, MAGED2*, and *ODF3L2* were mutated in all cluster 2 patients but not in any cluster 1 patients. Hence, these three genes were considered potential critical genes that might lead to different prognoses between the two clusters. This result differed from the traditional genotyping for detecting immunohistochemical levels of *CD10*, *Bcl-6*, and *MUM1* genes, indicating that our typing method may be different from the traditional typing in predicting prognosis (Figs. 2, 3).

*FTSJ3* (FtsJ RNA 2′-O-methyltransferase 3), located at chromosome 17q23.3, belongs to RNA methyltransferase (RNMT). *FTSJ3* has two homologues: *FTSJ1* and *MRM2* (*FTSJ2*). Studies have found that *FTSJ1* and *MRM2* have no effect on the in vivo screening of shRNA in most tumour cells [15, 16]; however, *FTSJ3* is commonly amplified and overexpressed in malignant tumours, such as breast cancer. *FTSJ3* is involved in the processing of 34S pre-rRNA to 18S rRNA and in 40S ribosomal subunit formation and is used by HIV-1 to evade innate immune recognition by IFIH1/MDA5 in cases of infection by HIV-1 virus [16]. Data in the COSMIC database (https://cancer.sanger.ac.uk/cosmic) demonstrate that the most common mutation type is missense substitution (47.44%) followed by synonymous substitution and in-frame deletion, accounting for 16.98 and 5.81%, respectively. Research by Manning et al. [17] illustrated that copy number amplification of *FTSJ3* was found in 6.26% of breast cancer patients. In addition, 1.26% of patients in the TCGA pan cancer cohort had *FTSJ3* mutations, and the mutation frequency was the highest (5.80%) in patients with uterine corpus endometrial carcinoma. *MAGED2* (MAGE family member D2) is a member of the *MAGED* gene family and is located on chromosome Xp11.2 [18]. According to Genecards (www.genecards.org/), the *MAGED2*-encoded protein is progressively recruited from the cytoplasm to the nucleoplasm during interphase and after nucleolar stress, thus affecting cell cycle regulation. *MAGED2* mutations can cause a form of transient antenatal Bartter’s syndrome. *MAGED2* mutations are also involved in several cancers, including breast cancer and melanoma. Papageorgio et al. [19] demonstrated that *MAGED2* interacted with *p53*, an important tumour suppressor gene, and changed its activity in human cultured cells. Although it is unclear whether *MAGED2* mutations affect tumour progression in lymphoma, studies from different fields confirmed that some of these gene mutations play a role in cell cycle progression and apoptosis [20]. *ODF3L2* (outer dense fibre of sperm tails 3-like 2) is located on chromosome 19p13.3, and diseases associated with *ODF3L2* include type 1 diabetes mellitus (T1DM) 15 and T1DM 5. The COSMIC database recorded 440 mutations in the *ODF3L2* gene, and these mutations are distributed in bladder cancer, colorectal cancer, liver cancer, lung cancer, thyroid cancer, and melanoma. At the time of writing, there is no related research on the role of the *ODF3L2* gene in the occurrence and development of cancer, suggesting that it may be a novel marker for the prognosis of DLBCL.

This study has the following limitations. First, the accuracy of this typing method needs to be further improved. This method is only suitable for DLBCL typing; in addition, the ability to correct PCR amplification bias and sequencing errors of NGS are unsolved problems in immune repertoire analysis. Expanding the sample size and including more pathological types are two scientific improvements. Second, due to the small number of patients, the traditional classification only divided patients into GCB and non-GCB groups rather than GCB, ABC and UC groups. Moreover, due to the small number of patients and short follow-up time, cluster 1 and GCB maintained 100% cumulative survival rates (Fig. 2). Therefore, the results may be biased, and the sample size needs to be expanded for in-depth research. Third, this method is expensive and time-consuming. Currently, this method has high cost and a long cycle (approximately 10 days). Therefore, this method is still far from clinical application, which requires not only the improvement of the typing methods but also progression of NGS technology.

## Conclusion

This study demonstrates the feasibility of a cluster typing model in DLBCL based on the BCR repertoire. Compared with the traditional immunohistochemistry classification, this method can predict the survival of DLBCL patients, especially after chemotherapy, more accurately. Mutational analysis preliminarily confirmed the association between the DLBCL subtype and specific mutant genes. *FTSJ3, MAGED2*, and *ODF3L2* may critical genes that explain the difference in prognosis between the two typing methods.

## Data Availability

The dataset used and/or analyzed during the current study are available from the corresponding author on reasonable request.

## Abbreviations

DLBCL: Diffuse large B-cell lymphoma
BCR: B-cell receptor
PFS: Progression-free survival
OS: Overall survival
NHL: Non-Hodgkin lymphoma
GEP: Gene expression profiling
GCB: Germinal center B cell
ABC: Activated B cell
TLR: Toll like receptor
NGS: Next-generation sequencing
IGH: Immunoglobulin heavy chain
FFPE: Formalin-fixed paraffin-embedded
CDR3: Complementarity determining region 3
GO: Gene Ontology
KEGG: Kyoto Encyclopedia of Genes and Genomes
UC: Unclassified subtype
IPI: International prognostic index
